# Voice Pitch Elicited Frequency Following Response in Chinese Elderlies

**DOI:** 10.3389/fnagi.2016.00286

**Published:** 2016-11-29

**Authors:** Shuo Wang, Jiong Hu, Ruijuan Dong, Dongxin Liu, Jing Chen, Gabriella Musacchia, Bo Liu

**Affiliations:** ^1^Otolaryngology – Head & Neck Surgery, Beijing Tongren Hospital, Beijing Institute of Otolaryngology, Capital Medical UniversityBeijing, China; ^2^Department of Speech-Language Pathology and Audiology, University of the Pacific, San FranciscoCA, USA

**Keywords:** aging-related, frequency following responses, Mandarin lexical tone, pitch processing, temporal processing

## Abstract

**Background:** Perceptual and electrophysiological studies have found reduced speech discrimination in quiet and noisy environment, delayed neural timing, decreased neural synchrony, and decreased temporal processing ability in elderlies, even those with normal hearing. However, recent studies have also demonstrated that language experience and auditory training enhance the temporal dynamics of sound encoding in the auditory brainstem response (ABR). The purpose of this study was to explore the pitch processing ability at the brainstem level in an aging population that has a tonal language background.

**Method:** Mandarin speaking younger (*n* = 12) and older (*n* = 12) adults were recruited for this study. All participants had normal audiometric test results and normal suprathreshold click-evoked ABR. To record frequency following responses (FFRs) elicited by Mandarin lexical tones, two Mandarin Chinese syllables with different fundamental frequency pitch contours (Flat Tone and Falling Tone) were presented at 70 dB SPL. Fundamental frequencies (f0) of both the stimulus and the responses were extracted and compared to individual brainstem responses. Two indices were used to examine different aspects of pitch processing ability at the brainstem level: Pitch Strength and Pitch Correlation.

**Results:** Lexical tone elicited FFR were overall weaker in the older adult group compared to their younger adult counterpart. Measured by Pitch Strength and Pitch Correlation, statistically significant group differences were only found when the tone with a falling f0 (Falling Tone) were used as the stimulus.

**Conclusion:** Results of this study demonstrated that in a tonal language speaking population, pitch processing ability at the brainstem level of older adults are not as strong and robust as their younger counterparts. Findings of this study are consistent with previous reports on brainstem responses of older adults whose native language is English. On the other hand, lexical tone elicited FFRs have been shown to correlate with the length of language exposure. Older adults’ degraded responses in our study may also be due to that, the Mandarin speaking older adults’ long term exposure somewhat counteracted the negative impact on aging and helped maintain, or at least reduced, the degradation rate in their temporal processing capacity at the brainstem level.

## Introduction

Many perceptual studies have shown that older adults with and without hearing loss may have difficulty with speech identification, especially in the presence of background noise (Dubno et al., 1984; Souza and Boike, 2006). Psychoacoustic studies have also found that in comparison with younger listeners, older adults with normal hearing may have more difficulty in using time-varying acoustic cues due to the deficits in temporal resolution (Moore and Glasberg, 1988; Moore et al., 1992; Tremblay et al., 2002, 2003; Harkrider et al., 2005). Temporal cues, including temporal envelope and fine structure, play dominant roles in speech perception and pitch perception (Rosen, 1992; Shannon et al., 1995; Smith et al., 2002; Xu and Pfingst, 2003; Wang S. et al., 2011). Thus, it has been proposed that speech identification difficulties suffered by older adults even with normal hearing may be attributed to their degraded temporal processing ability.

Neurophysiological and electrophysiological studies have also found evidence of delayed neural timing, decreased neural synchrony, and decreased temporal processing ability with age (Walton et al., 1998; Wang M. et al., 2011; Parbery-Clark et al., 2012). Tremblay et al. (2003) measured the cortical auditory evoked potentials (CAEPs) and observed that the N1 and P2 peak latencies recorded from older listeners were prolonged when they listened to the synthetic speech tokens. They proposed that the auditory neurons in older adults may need more time to recover from the initial excitation before firing again, so older adults may have decreased neural timing during transmission and processing auditory information. Age-related neural synchrony changes were not only observed by the use of auditory cortical potentials, but also reported by measuring auditory brainstem responses (ABRs) (Jerger and Hall, 1980; Konrad-Martin et al., 2012) and speech evoked ABRs (cABR) (Anderson et al., 2012). These studies reported common findings that the aging process reduces the amplitudes and increases the latency of ABR waves, suggesting degraded time-locked neural activity in the auditory brainstem of older listeners. Furthermore, Anderson et al. (2012) recorded ABRs to a speech syllable /da/ in both normal-hearing younger and older adults. They found that older adults had decreased phase-locking and smaller response magnitudes in comparison to younger adults, indicating that older adults have already had the deficits in temporal precision in the subcortical encoding of a speech sound.

The frequency following response (FFR) is a scalp-recorded measure that is dependent on the phase-locked brainstem activity in a population of neurons that phase-lock to the periodicity features in a sound (Krishnan and Parkinson, 2000; Krishnan, 2002; Krishnan et al., 2004; Aiken and Picton, 2008; Clinard et al., 2010). In a study, Krishnan et al. (2004) recorded human FFRs elicited by different Mandarin lexical tones. Mandarin lexical tones are characterized by syllable-level fundamental frequency (f0) contour patterns, including changes in pitch height and direction of the pitch contour. It was clearly shown that the phase-locked FFR activity carrying pitch-relevant information follows the pitch changes presented in each stimulus, suggesting that the representation of pitch changes in Mandarin tones are based on the temporal discharge patterns of phase-locked neural activity in the brainstem pathways. As older adults, even those with normal hearing, have shown degraded temporal processing with age-related distorted cortical neural activity, it will be interesting to see how the neural representation of pitch, as reflected by Mandarin tone elicited FFR, in older listeners preserve the pitch-relevant information in comparison with their younger counterparts. By this date, few studies have investigated how aging may impact the neural representation of pitch in the brainstem using the FFR recording measure, especially in populations that speak a tonal language. Clinard et al. (2010) examined the relationship between behavioral performance, as measured by a frequency discrimination task, and physiological performance, as measured by FFRs, using the same 500 and 1000 Hz tone bursts. They found that as the age of listeners increased, pitch discrimination ability and neural representation of frequency both decreased, but no significant relationship was found between each other.

The present study was aimed at investigating the neural representation of Mandarin lexical tones in the brainstem of Mandarin-speaking older adults with normal hearing using the FFR technique. It is hypothesized that in comparison with the younger adults, older adults may have less faithful representations of the f0 contours in lexical tones and decreased robustness of neural phase-locking.

## Materials and Methods

### Ethics Statement

Experimental protocols and procedures used in this study were approved by the Beijing Institute of Otolaryngology Institutional Review Board.

### Participants

**FIGURE 1:**
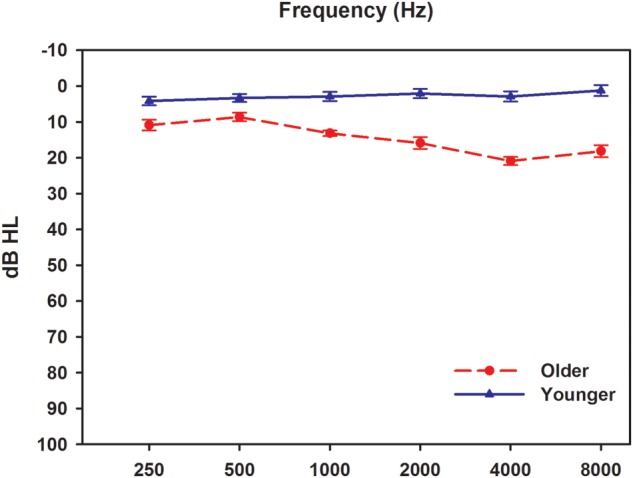
**Audiometric results from two groups.** Results were obtained using GSI 61 audiometer with supra-aural TDH headphones. Solid and dashed lines indicate younger and older adults, respectively. Error bars represent standard errors.

Twelve younger participants and 12 older participants were recruited in this study. The younger participants were aged 22–25 years old (mean ± SD = 22.9 ± 0.8), while the older participants were aged 61–69 years old (mean ± SD = 62.3 ± 2.8). All participants, 13 males and 11 females, were native speakers of Mandarin Chinese. Before each test session, all participants underwent a complete audiological assessment evaluation to fulfill the requirement of hearing sensitivity ≤25 dB HL at octave frequencies from 125 to 8000 Hz (**Figure 1**). The audiological assessments included otoscopy, pure tone audiometry (using a GSI-61 clinical audiometer), tympanometry (using a GSI 33 Middle ear analyzer), and ABRs test. The ABRs were recorded using the Smart EP module in the IHS System. The click stimuli were presented monaurally at an intensity starting at 80 dB nHL with a rate of 11.1/s. A total of 2,048 sweeps were recorded using two polarities, rarefaction and condensation. All participants had within normal range wave V latencies (5.70–6.08 ms; 5.88 ± 0.16 ms) in the click-evoked ABR tests. Experimental protocols and procedures used in this study were approved by the Beijing Tongren Hospital Institutional Review Board and the Ethical Committee. All participants were informed of the purpose and content of the study, and signed an informed consent form. All data collection was conducted in a sound-proof booth at the China Rehabilitation Research Center for Deaf Children.

### Recordings

Two monosyllabic Mandarin Chinese syllables with contrast pitch contours (Flat Tone, ‘clothes’ and Falling Tone, ‘meaning’) were prepared. These voice samples then underwent a temporal normalization to a unified duration of 250 ms with a rising/falling time of 10 ms. The final stimulus tokens of the two Mandarin syllables had the frequency ranges containing their f0 contours ranging from 163 to 180 Hz for Flat Tone, and 105–156 Hz for Falling Tone. Stimulus tokens were controlled and delivered via Stim2 system. They were presented monaurally to the participant’s right ear through a custom-built electromagnetically shielded insert earphone modified from an ER3-A earphone (Bio-logic Systems, Corp., Mundelein, IL, USA) at 70 dB SPL. Each token was presented for blocks of 2000 accepted sweeps, where artifact rejection criteria was set at ±25 μV. The inter-stimulus interval between adjacent stimulus tokens was set at 45 ms and the tokens were presented in a random order within and across participants. A control condition (sound tube occluded and removed from the participant’s ear) was conducted at the end of each recording session to ensure that the stimulus artifact was eliminated from the recordings.

Three gold-plated recording electrodes were applied to each participant on the forehead (Fpz, non-inverting), right mastoid (M2, inverting), and left mastoid (M1, ground). All electrode impedances were maintained under 3 kOhms at 10 Hz. Continuous recordings, collected by NeuroScan ACQUIRE 4.4., were amplified using the SynAmp 2 (Compumedics, Compumedics, VIC, Australia) system with a 24-bit resolution and a least significant bit of 0.15 nV.

### Analyses

All the data was analyzed using MatLab (Mathworks, Natick, MA, USA) and EEGLab (Swartz Center for Computational Neuroscience, San Diego, CA, USA). Recorded EEGs were filtered through a 100–1500 Hz band-pass filter with a linear-phase of 500 poles. The artifact rejection criteria was set at ±25 μV. After averaging the remaining EEG sweeps, a cross-correlation between the stimulus and recorded waveforms was carried out to identify the time shift data point that had the maximum cross-correlation value between the 2–10 ms response window. Starting from this data point, a segment of 250 ms was extracted from the averaged data.

A fundamental frequency extraction algorithm based on narrowband spectrogram (Jeng et al., 2011a) was used to extract the pitch information of the sampled EEG signal. In short, all stimulus tokens and recordings were first segmented using a 50 ms sliding Hanning window with a step size of 1 ms. Within each windowed segment, the frequency corresponding to the maximal peak of the spectral amplitudes within a predefined frequency range is determined as the f0 frequency for that segment. This procedure was repeated for all windowed segments.

Two indices, namely Pitch Correlation and Pitch Strength, were used to quantify the pitch-tracking accuracy and phase-locking magnitude of the responses. They each represent a different aspect of pitch processing in the brainstem. Pitch Correlation describes how close the f0 of the responses are correlated to that of the stimulus. Using the above mentioned f0 extracting technique, the f0 of both the responses and stimulus were extracted and the correlation coefficient between them was denoted as the Pitch Correlation index. It describes how precise the brainstem’s response tracks the pitch change in the stimulus. Pitch Strength was used to quantify the phase-locking magnitude of the responses (Krishnan et al., 2005; Jeng et al., 2010; Xu and Jeng, 2011). This index was derived from an autocorrelation function that allowed the measurement of overall periodicity of a sampled signal. Specifically, each recording (i.e., the entire 250 ms of a recording) was multiplied by a duplication of itself with increasing time shifts. For each time shift, an autocorrelation value was calculated and expressed between -1 and 1. F0 was calculated using the output of the autocorrelation function by finding the time shift that yielded the maximum autocorrelation value and taking the inverse of that time shift. Because the f0 contour of the stimulus token used in this study fell within the frequency range of 100–200 Hz, the time shifts were limited to 5–10 ms when searching for the location of the maximum peak in the autocorrelation output. Pitch Strength was calculated using the autocorrelation function by finding the peak-to-trough amplitude starting from the maximum positive peak (within the 5–10 ms time shifts) to the following negative trough in the normalized autocorrelation output. Between the two indices, Pitch Correlations emphasize how faithful the brainstem response is to the stimulus, where Pitch Strength focuses on the magnitude of the response itself. A Student’s *t*-test was carried on each index for comparisons between the younger and older adult groups for statistical analysis.

## Results

**FIGURE 2:**
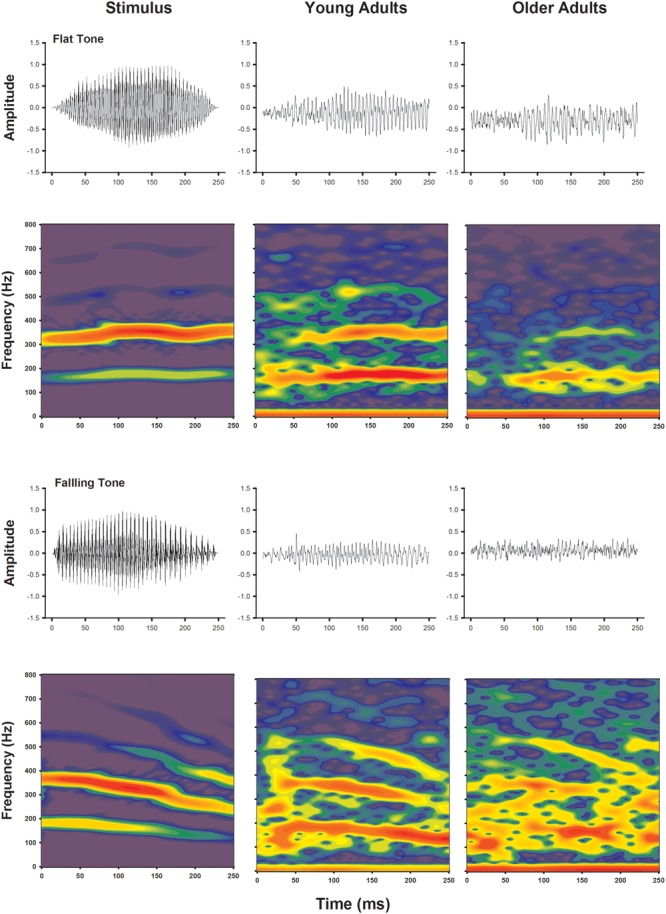
**Temporal waveforms (upper rows) and spectrograms (lower rows) of the stimuli (left columns), group averaged responses from the younger adults (middle column) and older adults (right columns)**.

Frequency following response to voice pitch was visualized by plotting waveforms and spectral energies of the recordings as a function of time. Waveforms and spectrograms of the voice pitch elicited FFR obtained from grand averages of both younger and older adults are shown in **Figure 2**, for Flat Tone and Falling Tone, respectively. Waveforms obtained from both age groups demonstrated clear periodicity and change of periodicity that mimics the fundamental frequencies of the stimuli and their temporal contours. Similarly, spectrograms of the recordings of both age groups also showed clear FFR energy that followed that of the stimulus. The FFR energy following the harmonics was not as clear.

**FIGURE 3:**
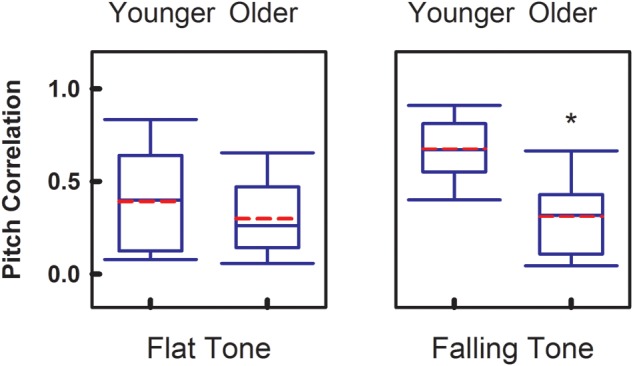
**Group comparison of Pitch Correlation between younger adults and older adults, elicited by Flat Tone **(left)** and Falling Tone **(right)**.** Boundaries of the boxes indicate the 25th and 75th percentile. Whiskers indicate the 10th and 90th percentiles. Solid lines denote the median. Dashed red lines denotes the mean. Error bars represent standard errors. Asterisk denotes significant group difference between groups (*p* < 0.05).

To compare the overall faithfulness of the neural responses in the brainstem to the stimulus, **Figure 3** demonstrates the group comparisons of Pitch Correlations between age groups for both Flat Tone and Falling Tone. Significant group difference (*p* < 0.05) of Pitch Correlation was found in Falling Tone, which has a falling pitch contour, between the younger (*r* = 0.67 ± 0.16) and the older group (*r* = 0.31 ± 0.21). Pitch Correlations obtained from the younger group (*r* = 0.39 ± 0.27) were greater, yet not significantly different, from that of the older group (*r* = 0.30 ± 0.20) when Flat Tone was used as the stimulus.

**FIGURE 4:**
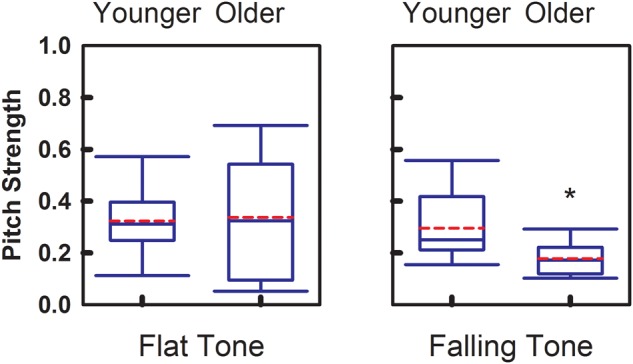
**Group comparison of Pitch Strength between younger adults and older adults, elicited by Flat Tone **(left)** and Falling Tone **(right)**.** Boundaries of the boxes indicate the 25th and 75th percentile. Whiskers indicate the 10th and 90th percentiles. Solid lines denote the median. Dashed red lines denotes the mean. Error bars represent standard errors. Asterisk denotes significant group difference between groups (*p* < 0.05).

Magnitude of the phase-locking mechanism at the brainstem level is represented by the Pitch Strength index. **Figure 4** demonstrates the group comparisons of Pitch Strength obtained from the two age groups. Similar to Pitch Correlation, Falling Tone yielded significantly greater (*p* = 0.017) Pitch Strength in the younger adult group (*r* = 0.30 ± 0.14) than that obtained from the older group (*r* = 0.18 ± 0.07). Pitch Strength obtained from the younger group (*r* = 0.32 ± 0.14) for Flat Tone was not significantly different from that from the older group (*r* = 0.33 ± 0.25).

## Discussion

Overall, results of this study demonstrated that in a tonal language speaking population, the pitch processing ability at the brainstem level in older adults are not as strong and robust as their younger counterparts. This is not surprising given that the temporal processing capacity of older adults are generally degraded due to aging (Wingfield et al., 1999; Tremblay et al., 2002). Previous electrophysiological reports have also provided objective evidence that the aging process affects the neural processing functions along the auditory pathway (Jerger and Hall, 1980). For example, compared to younger adults, older adults have prolonged latencies in click evoked ABR when a higher presentation rate was used (Burkard and Sims, 2001). As to speech evoked brainstem responses, such as Consonant-Vowel (CV) evoked cABR, evidence has found that normal hearing older adults have diminished neural precision. (Anderson et al., 2012, 2013a,b).

In this study, Mandarin voice pitch elicited FFR obtained from younger Mandarin speaking adults were significantly stronger than that of older adults in Falling Tone, but not Flat Tone, which has a flat f0 contour. This is consistent with previous Mandarin tone elicited FFR reports (Krishnan et al., 2004; Jeng et al., 2010, 2011b) where in normal hearing young adults, Falling Tone usually elicited stronger FFR than Flat Tone. It was suggested that the change of f0 contour of Falling Tone, which started around 180 Hz and falls to around 120 Hz in just 250 ms, requires additional neural encoding in the brainstem area to track such rapid pitch change. The results from our study may suggest that such ability to encode rapid spectral change in voice pitch, e.g., Mandarin Falling Tone, may have degraded in the older group, while the ability to detect and follow relatively flat pitch, e.g., Flat Tone, remained fairly stable in the older group, when compared to their younger counterparts. This finding is also consistent with previous reports on cABR in older populations that did not speak tonal languages (Anderson et al., 2012, 2013a,b). In those studies, older adults’ responses were notably poorer during rapid spectral transitions, such as transition between a transient consonant and a steady-state vowel, which is similar to the results of our study where change of spectral slope made it harder for older adults to track.

On the other hand, auditory exposure and trainings have been proven to have a positive impact on behavioral auditory processing capacity (Kishon-Rabin et al., 2001; Micheyl et al., 2006; Strait and Kraus, 2014), as well as enhance auditory electrophysiological responses, including brainstem and cortical responses (Marques et al., 2007; Musacchia et al., 2007, 2008; Marie et al., 2010; Chobert et al., 2011). Specifically for scalp reported FFR, reports have shown that due to neural plasticity at the brainstem level, short-term and long-term auditory exposure, including music training and language exposure, could enhance FFR responses in different age groups with different language backgrounds (Krishnan, 2002; Krishnan et al., 2004; Musacchia et al., 2007; Jeng et al., 2010, 2011b). In this study, as our older adult groups are on average nearly 40 years older than their younger counterparts, the length of their exposure to a tonal language are notably longer too.

From this perspective, since both aging and length of language exposure play a part in reshaping the neural pathway, it may be difficult to discern which one is the dominating factor here. However, reports from cABR in older adults have shown that without lifelong exposure to a language that is acoustically rich in pitch information, older adults almost always showed significantly declined brainstem responses to pitch (Anderson et al., 2012, 2013b). In our study, Falling Tone elicited significantly stronger responses in the younger adults than that of older adults, while Flat Tone did not. Thus, it may be possible that the Mandarin speaking older adults’ long-term exposure somewhat counteracted the negative impact on aging and helped maintain, or at least reduced, the degradation rate in their temporal processing capacity at the brainstem level.

Findings from this study could provide insight to current research and clinical applications of auditory training. For example, it is well-established that short-term and long-term auditory training, especially music training, can provide significant enhancement in pitch processing, speech in noise performance and other behavioral responses (Parbery-Clark et al., 2011, 2012; Alain et al., 2014), as evidenced by electrophysiological studies (Musacchia et al., 2007, 2008; Strait et al., 2012; Anderson et al., 2013a). Similar to lifelong music training, language exposure across the life span could be as analogous to a long-term auditory training program. The maintenance of excitatory and inhibitory subcortical neural networks (Parbery-Clark et al., 2012) for the perception of tonal information is essential for Mandarin speaking populations and may have been executed during this lifelong auditory training. It may suggest that long-term auditory training can improve and enhance neural phase-locking ability at the brainstem level, which will be beneficial for the pitch encoding capacity.

For future directions, we will continue monitoring the status and change of pitch coding mechanism at the brainstem level with the aging process. With a better understanding of the aging factor in neural encoding at brainstem and the associated plasticity characteristics, we can associate it with behavioral studies that focus on temporal processing in the same population and broaden our understanding of the aging process as a whole.

## Author Contributions

Conceived and designed the experiments: SW, JH, and BL. Performed the experiments: SW, RD, DL, and JC. Analyzed the data: SW, JH, and GM. Contributed reagents/materials/analysis tools: SW and JH. Wrote the paper: SW, JH, and BL.

## Conflict of Interest Statement

The authors declare that the research was conducted in the absence of any commercial or financial relationships that could be construed as a potential conflict of interest.
